# Implementation of the Surgical Safety Checklist in Switzerland and Perceptions of Its Benefits: Cross-Sectional Survey

**DOI:** 10.1371/journal.pone.0101915

**Published:** 2014-07-18

**Authors:** Stéphane Cullati, Marc-Joseph Licker, Patricia Francis, Adriana Degiorgi, Paula Bezzola, Delphine S. Courvoisier, Pierre Chopard

**Affiliations:** 1 Quality of care service, University Hospitals of Geneva, Geneva, Switzerland; 2 Division of anesthesiology, University Hospitals of Geneva, Geneva, Switzerland; 3 Quality of care unit, Ente Ospedaliero Cantonale, Lugano, Switzerland; 4 Patient Safety Foundation Switzerland, Zürich, Switzerland; 5 Division of clinical epidemiology, University Hospitals of Geneva, Geneva, Switzerland; University of Southern California, United States of America

## Abstract

**Objectives:**

To examine the implementation of the Surgical Safety Checklist (SSC) among surgeons and anaesthetists working in Swiss hospitals and clinics and their perceptions of the SSC.

**Methods:**

Cross-sectional survey at the 97^th^ Annual Meeting of the Swiss Society of Surgery, Switzerland, 2010. Opinions of the SSC were assessed with a 6-item questionnaire.

**Results:**

152 respondents answered the questionnaire (participation rate 35.1%). 64.7% respondents acknowledged having a checklist in their hospital or their clinic. Median implementation year was 2009. More than 8 out of 10 respondents reported their team applied the Sign In and the Time Out very often or quasi systematically, whereas almost half of respondents acknowledged the Sign Out was applied never or rarely. The majority of respondents agreed that the checklist improves safety and team communication, and helps to develop a safety culture. However, they were less supportive about the opinion that the checklist facilitates teamwork and eliminates social hierarchy between caregivers.

**Conclusions:**

This survey indicates that the SSC has been largely implemented in many Swiss hospitals and clinics. Both surgeons and anaesthetists perceived the SSC as a valuable tool in improving intraoperative patient safety and communication among health care professionals, with lesser importance in facilitating teamwork (and eliminating hierarchical categories).

## Introduction

In Switzerland, over 1 million operations are carried out annually and major complications are expected to occur in 3 to 20% of patients with a mortality rate between 0.8 and 1.5% [1]. Although surgical procedures are performed to save lives and to improve patient's quality of life, unsafe practice and medical errors have also been incriminated in causing serious complications. Such preventable complications have been estimated to increase the total hospital cost by an average 10% [2].

With the increasing burden of patients' comorbidities [3]–[6], the complexity of the surgical operation and application of advanced technologies, the achievement and maintenance of clinical excellence has become increasingly challenging. Many lessons have been learned from the aviation industry where checklists and training in crew resource management have largely contributed to decrease the incidence of accidents and strengthen safety culture [7]–[9].

In 2007, the World Health Organization (WHO) initiated the Safer Surgery Saves Lives project [10], in which standards for the safe delivery of surgical care and a 19-item Surgical Safety Checklist (SSC) were developed [11]. This SSC encompasses the three phases of any operation that mirror the take-off cruise and landing phases of the aviation-industry. Before anaesthesia induction (“sign in”), the team confirms patient identity, surgical site, anaesthetic concerns, allergies and estimated surgical blood losses. Before skin incision (“time out”), the entire team is introduced, the anticipated critical events are reviewed, sterility and antibiotic administration is confirmed, and, imaging and other diagnostic elements are displayed if appropriate. Before the patient leaves the operating room (OR) (“sign out”), the swab count is confirmed, procedure name and handling of specific tissue/fluid specimen is confirmed and, equipment, postoperative treatment and patient destination are addressed. This SSC was trialled in eight different countries, representing a wide spectrum of health care systems and environments. Using a pre-post-design and including more than 8'000 patients, the implementation of the SSC resulted in a 30% reduction in operative mortality and major complications, in both high and low income countries [12]. A meta-analysis confirmed the effectiveness of the SSC for mortality (relative risk (RR) 0.57, 95% confidence interval (CI): 0.42–0.76) and complications (RR 0.63, 95% CI 0.58–0.67) [13], however the risk of bias remains elevated considering the large number of low quality studies, as noted in two systematic reviews [13], [14].

Acknowledging the positive impact the SSC could have on patient safety, the UK National Patient Safety Agency issued an alert stating that the SSC (in its modified version) should be completed for every surgical patient from February 2010 onwards [15]. Likewise, national health regulatory authorities in France [16] and Ireland [17] endorsed the mandatory use of SSC for all patients undergoing surgery. In other Western countries, professional health organisations for surgical care have recommended the routine utilization of SSC [18]. In Switzerland, the Patient Safety Foundation has dealt with safety issues and quality of care since 2003 and a recent survey among directors of surgery departments reported that the SSC was used in 74% of hospitals [19]. However, despite an “official” adhesion to SSC of the majority of perioperative health care professionals [20]–[23], there are many barriers hindering the success of its implementation [24] and the promotion of a true safety culture [20], [22], [23], [25]–[30]. A recent survey among Swiss surgical healthcare professionals showed moderate satisfaction with the SSC [31]. This mixed picture suggests that the implementation of the checklist is facing not only barriers from healthcare organizations but also barriers related to the perception of the benefit of the SSC. We know little about perceptions of the SSC among Swiss surgeons and anaesthetists.

The objective of this study was twofold: to report on the initial implementation of SSC among Swiss hospitals and to describe personal opinions of surgeons and anaesthetists towards the SSC.

## Methods

### Setting and design

The study was initiated in 2010 by a multidisciplinary team including anaesthetists, surgeons, a psychologist, a sociologist and clinical quality officers from the University Hospital of Geneva, in collaboration with the Swiss Patient Safety Foundation in Zurich. A comprehensive questionnaire was developed to address various aspects of the SSC: its implementation in the respondent's hospital/clinic, self-reported compliance and general perceptions of the SSC. The questionnaire was designed to provide a comprehensive view of the application and the attitude towards the SSC among practicing Swiss surgeons and anaesthetists. The questionnaire was translated in German, French and Italian. Each translation was conducted by two native translators and pre-tested.

The questionnaire was distributed to all participants of the joint meeting of the Swiss Society of Surgery and the Swiss Society of Anaesthesia and Reanimation that was held during the 97^th^ Annual Meeting of the Swiss Society of Surgery in Interlaken in June 2010 (26^th^–28^th^). The purpose of the cross-sectional survey was addressed in an explanatory letter and presented by the chairman of the plenary lecture. Questionnaires were distributed by bilingual medical students, along with a Swiss chocolate to attendees agreeing to participate. No consent was asked to an Institutional Review Board because the survey was administered during a congress and no identifying information was asked to respondents.

### Variables of interest (outcome)

#### Implementation of the SSC

The implementation of the SSC was addressed by asking participants whether the SSC was applied in their institutions (yes, no, don't know, I don't know this surgical safety checklist), whether they received support from their administrative and clinical managing authorities (no, little, medium, strong, or very strong support) and whether different sections – Sign In, Time out, Sing Out, Others – were included in their SSC (yes, no),

#### Perceived compliance to the SSC

The SSC's use was addressed with the following question: “please indicate if these checklist sections are applied either never (0%), rarely (1–29%), partially (30–59%), very often (60–90%), or quasi systematically (>90%) within your surgical/anaesthetic team (i.e., the one with which you operate most often)”. Space was also allocated for free commentary.

#### Perception of the SSC

Based on the literature assessing the perception of the checklist, a list of 8 questions was built to assess the general perceptions of the SSC. Respondents had to report their agreement with each item on a scale ranging from 1 (don't agree at all) to 5 (fully agree) with an additional “No opinion” option.

### Descriptive variables

The first question addressed the sector of employment (public or private) where the SSC was implemented. Sociodemographic and professional characteristics of the participants included sex, age (year of birth), profession (surgeon, anaesthetist), postgraduate training abroad (“After completing your specialist training, have you followed part of your postgraduate training in another country”? yes vs. no), type of employment (private or public sector; University or non-University hospital), workload (number of interventions performed each year) and clinical experience (number of years of clinical practice in the specialty).

### Statistical analysis

Given the exploratory nature of this cross-sectional survey, no power calculation was conducted. Characteristics are presented as frequencies, means, standard deviations and medians as appropriate. To describe physician's attitude and perception regarding the SCC, the 5-point Likert scale was collapsed into a 3-point scale (positive, neutral, negative). We examined sub-groups differences with t-test if the outcome was continuous and with Chi-square test if the outcome was ordinal or dichotomous. In case of small sample size in sub-groups, we used the Fischer exact test. When a continuous outcome was not normally distributed, we used the Wilcoxon-Mann-Whitney test. The level of significance was set at p<0.05 and all tests were two-sided. All statistical analyses were completed using the SPSS software (version 18; SPSS Inc, Chicago, IL).

## Results

### Participants

Out of 433 questionnaires distributed at the meeting, 152 were returned (response rate 35.1%). As described in Table 1, the majority of respondents were men (62.6%), German speaking (84.2%), surgeons (61.6%) and employed in a public hospital (78.4%) with a median of 500 surgical procedures being performed annually. Their mean age was 44.5 years and, on average, they had been in practice for 16 years.

**Table 1:** Characteristics of participating physicians (N = 152).

	N (%)^a^
Sex (N = 139)	
Men	87 (62.6)
Age [year] (N = 135)	
Mean age (SD)	44.5 (11.1)
Range	20–79
*by age classes:*	
20–37	48 (35.6)
38–50	45 (33.3)
51 and older	42 (31.1)
Profession (N = 138)	
Surgeon	85 (61.6)
Anesthetists	47 (34.1)
Others	6 (4.3)
Number of year of practice (N = 116)	
Mean (SD)	15.6 (10.1)
Range	1–40
Number of interventions/procedures by year (N = 102)	
Median (Mean, SD)	500 (833, 1325)
Range	20–8000
Type of employment (N = 139)	
Public sector	109 (78.4)
* non-university hospital*	*91 (83.5)*
* university hospital*	*18 (16.5)*
Private sector	30 (21.6)
*private practice*	*21 (70.0)*
*private hospitals/clinics*	*9 (30.0)*
Postgraduate training in another country (N = 121)	
yes	41 (33.9)
no	80 (66.1)
Language of the questionnaire	
German	128 (84.2)
French	23 (15.1)
Italian	1 (0.7)

a Total of percentage exceed 100% due to surrounding.

### Implementation of the SSC

At the time of the survey, a SSC similar to the one proposed by the WHO was used by 65% of the respondents, the implementation being slightly higher in the public health sector than in the private sector (73% vs. 56%, respectively; p = 0.048). The median year of SSC adoption was 2009 (2007 and before, n = 5; 2008, n = 19; 2009, n = 44; 2010, n = 16; missing values, n = 68).

The implementation of the SSC within hospitals and clinics was supported by all institutional health care authorities (general management, surgery, anaesthesiology and nursing managers), although participants noted stronger support from anaesthesia departments and less support from nursing departments (Table 2). No differences were observed between the private and the public sectors.

**Table 2:** Institutional support to the implementation of the Surgical Safety Checklist (Switzerland, 2010).

*“In your opinion, to what extent was the implementation of the checklist supported by…”*	No support or little support	Medium support	Strong support or very strong	Mean* (SD)
the chief executive management in your hospital/clinic (N = 73)	6.8%	15.1%	78.1%	4.27 (0.96)
the chairman and consultants of the department of surgery (N = 89)	5.6%	19.1%	75.3%	4.24 (0.95)
the chairman and consultants of department of anesthesiology (N = 86)	3.5%	5.8%	90.7%	4.56 (0.81)
the head nursing department and operating theater manager (N = 81)	22.2%	11.1%	66.7%	3.84 (1.27)

* No support  =  1, Little support  = 2, Medium support = 3, Strong support = 4, Very strong support = 5.

### Perceived compliance to the SSC

As shown in Table 3, the “sign in” (91.5%) and “time out” (96.0%) sections of the SSC were largely included in the checklist used by respondents in their hospitals/clinics. According to respondents, the “sign in” and “time out” were completed “very often or quasi systematically” (89.9% and 82.5%, respectively). In contrast, the “sign out” was included only half the time in the checklist, and 45.2% of respondents described it as “never or rarely” completed. The proportion of “never or rarely” applied differed between the private and the public sectors (17% vs. 61%, respectively; p = 0.012). Additional sections to the WHO SCC were reported by 27% respondents, with no differences being observed between the private and the public sectors.

**Table 3:** Content of the Surgical Safety Checklist (SSC) implemented in the hospital's respondent and perceived compliance rate to the SSC (Switzerland, 2010).

	Context of the SSC implemented in the hospital's respondent:	Perceived compliance rate to the SSC^a^				
	%	Never (0%) or rarely (1–29%)	Partially (30–59%)	Very often (60–90%) or quasi systematically (>90%)	Mean* (SD)	Missing
Sign In (N = 94)	91.5%	4.5%	5.6%	89.9%	4.6 (0.9)	1
Time Out (N = 101)	96.0%	8.2%	9.3%	82.5%	4.4 (1.0)	4
Sign Out (N = 87)	49.4%	45.2%	8.2%	46.6%	3.0 (1.8)	14
Other section (N = 45)	26.7%	10.0%	0%	90.0%	4.6(1.0)	2

a “Please indicate if these checklist sections are applied either never, rarely, partially, very often, or quasi systematically within *your surgical/anaesthetic team* (i.e., the one with which you operate most often)”.

* Never = 1, Rarely = 2, Partially = 3, Very often = 4, Quasi systematically = 5.

### Opinions regarding the SSC and its impact

The majority of respondents agreed that the SCC was a valuable tool for improving intraoperative safety (89.5%), developing a safety culture among surgical teams (75.4%) and fostering team communication (68.8%) (Table 4). In line with these perceptions, respondents largely disagreed with the opinion that the SCC is “a waste of time” (68.8%), “brings no added value to existing safety procedures” (61.5%) and “has not demonstrated its efficacy in the scientific literature” (53.7%). Opinions were less positive for the items “SSC facilitates teamwork” and “eliminates hierarchy (during the controls) between healthcare professionals”(43.3% disagreed).

**Table 4:** Attitudes toward the Surgical Safety Checklist (Switzerland, 2010).

*The checklist…*	Don't agree at all or don't agree	Partially agree	Fully agree or agree	Mean* (SD)
improves the safety of procedures (anaesthetic and surgical) (N = 143)	5.6%	4.9%	89.5%	4.5 (0.9)
is a waste of time (N = 138)	68.8%	15.9%	15.2%	2.1 (1.2)
improves team communication (related to safety) (N = 141)	12.1%	19.1%	68.8%	3.8 (1.1)
brings no extra value to existing safety procedures already in place in my hospital/clinic before its implementation (N = 130)	61.5%	20.8%	17.7%	2.3 (1.2)
helps to develop a safety culture in surgical teams (N = 138)	10.1%	14.5%	75.4%	4.0 (1.1)
has not demonstrated its efficacy in the scientific literature (N = 95)	53.7%	24.2%	22.1%	2.4 (1.3)
facilitates teamwork (N = 136)	21.3%	33.1%	45.6%	3.4 (1.1)
eliminates (during the controls) hierarchy between healthcare professionals (N = 127)	43.3%	31.5%	25.2%	2.7 (1.2)

*Don't agree at all = 1, Don't agree = 2, Partially agree = 3, Agree = 4, Fully agree = 5.

Respondents who did not use the SCC tended to express more negative opinions regarding the SCC (compared to respondents using the SSC). The SSC was more often significantly perceived as “a waste of time”, “bringing no extra value to existing safety procedures”, and having “not demonstrated its efficacy in the scientific literature” (Table 5). The SCC was also more frequently perceived as “a waste of time” in the private sector than in the public sector. Age, gender, clinical experience, and training abroad did not influence judgement towards the SCC (data not shown). Likewise, attitudes towards the SCC among surgeons and anaesthetists were similar, except that anaesthetists agreed more often that the SCC eliminates interprofessional hierarchy (Table S1).

**Table 5:** Attitudes towards the SSC among respondents working in hospitals with or without the SSC and those working in the public or private sector (Switzerland, 2010).

	Respondents working in hospitals					
*Proportion of “Fully agree or agree” with the following items: The checklist…*	with checklist (N = 97)	without checklist (N = 48)	p-value*	public hospitals (N = 109)	private hospitals (N = 30)	p-value*
improves the safety of procedures	91.7%	86.7%	0.556	90.6%	84.0%	.469
is a waste of time	10.8%	23.3%	0.005	9.9%	36.0%	.003
improves team communication	73.4%	60.0%	0.140	67.0%	69.2%	.828
brings no extra value to existing safety procedures already in place in my hospital/clinic	14.0%	23.8%	0.020	16.7%	24.0%	.394
helps to develop a safety culture in surgical teams	70.7%	86.4%	0.493	71.6%	84.6%	.215
has not demonstrated its efficacy in the scientific literature	16.4%	31.3%	0.030	19.4%	31.6%	.347
facilitates teamwork	45.6%	47.7%	0.816	43.0%	44.0%	.928
eliminates hierarchy between healthcare providers	24.7%	27.5%	0.197	24.7%	26.1%	.893

* Chi-square test or Fisher exact test if at least one cell had a frequency of 10 or less.

## Discussion

Since 2007, the implementation of the SSC has achieved relative worldwide success [16], [21], [32], [33] and the objective of this study was to explore the situation in Switzerland and to answer a burning question: Is Switzerland keeping up? We found that 65% of respondents were already using a SSC in their hospital/clinic in the year of 2010, an encouraging result, suggesting that a majority of Swiss hospital and clinics are convinced of the importance of the SSC. Another Swiss survey conducted one year later (2011) found an implementation rate of 74% [19], but used another design for the questionnaire administration and was conducted among another population (directors of adult departments in operative medicine); however, this survey obtained a response rate (30%) similar to ours. More recently, a survey conducted in December 2012 among members of the Foederatio Medicorum Chirurgicorum Helvetica found an implementation rate of 79% (of a surgical checklist based on the WHO-checklist, on the Universal Protocol or other) [31], but was also impeded by a low response rate (23%). These results clearly indicate that the implementation of the SSC has evolved favourably in Switzerland; however, the low participation rate, potentially causing bias, stresses the need for additional research in this area.

Importantly, the median year of implementation was 2009. Considering that the SSC was launched worldwide in 2007 and in Europe in January 2009 [34], this suggests that most Swiss hospital and clinics have been responsive within a very short time to the campaign of “Safer Surgery Saves Lives”.

Another encouraging result is the perceived positive reaction of hospitals' authorities towards the implementation of the SSC. Indeed, institutional support was seen as globally positive, across all kinds of authorities (management, surgery, anaesthesiology, and nursing). At most, we observed differences between perceptions of managerial support in anaesthesia and nursing. In anaesthesia, the support was perceived as strong or very strong by more than nine out of ten respondents, while in nursing it was “only” two out of three. Most notably, we did not find significant differences between private and public healthcare institutions. Comparison of this result with existing literature is limited as they are few studies examining institutional support of SSC's implementation. We found only one French study showing that the SSC was supported by medical department and management in more than 80% [35].

### Perceived compliance to the SSC

Most of the international literature about the compliance to the SSC in high-income countries show a relative heterogeneity: self-reported compliance rates vary between 38% and 96% [20], [23], [28], [30], [36], whereas administrative audits have shown compliance rates between 66% and 100% [26], [27], [37]–[40] and observational studies have shown that the SSC was initiated between 80% and 99% [24], [41]–[45]. But most of these compliance studies assessed the overall compliance without distinguishing the three parts of the SSC (Sign In, Time Out and Sign Out). Our results went further by assessing the compliance for each section and showed different perceived rates: almost 90% of respondents rated the SSC being applied by their surgical/anaesthetic team as “very often or quasi systematically” for the Sign In and 83% for the Time Out; however, the compliance with the Sign Out was rated applied “very often or quasi systematically” by only 47% of respondents. In other words, when implemented in hospitals and clinics, the SSC seems differently applied according to its content: before skin incision (Sign In and Time Out), the SSC seems conscientiously followed by both surgical and anaesthetic teams. However, at the end of the interventions, the compliance rate is perceived lower, a result similar to observational studies [24], [46]. This lack of compliance with the Sign Out could be rooted in the different perceptions of when to initiate this phase (toward the end of the procedure, the surgeon operator could ask to initiate the Sign Out, although the “last stitch” and the dressing might not have been completed), as noted by an observational study of team interactions during the application of the SSC [24].

Most notably, we observed no difference in compliance between the private and the public sectors. This result is discordant with a study conducted in a central American country [47], where the compliance rate was higher among private hospitals.

Although the compliance rate reported in the current study is encouraging, it should be noted that routine could reduce it over time. Indeed, a qualitative observational study assessed the compliance rate to 5 new items into the checklist already in use in an healthcare organisation and results showed that compliance rate with the introduced items was high (between 75 and 86%) –a result similar to studies examining the compliance in settings where the checklist was recently implemented [24], [41]–[45] – while compliance with the existing (well-established) items was relatively low (between 12% and 72%) [28].

### Perceptions of the SSC

Opinions of the respondents toward the SSC are generally positive. The SSC is perceived as an instrument, that could improve patient safety in hospitals, a result similar to other studies [20], [21], [23], [48].

Some studies have pointed out that time pressures were a hindrance to compliance with the SSC, for examples in US [49] and French [26] contexts. In Switzerland, our study revealed that the SSC was not perceived as a waste of time. This is confirmed by observational studies which found that checking the items of the SSC is not time-consuming: the duration of the Time Out phase was between mean of 36[s] [24] and median of 60[s] [46].

Another barrier reported in the literature pointed out that some items of the SSC could unnecessarily duplicate existing procedures of control [26]. In our survey, more than six out of ten respondents disagreed with the opinion that the SSC brought no extra values to existing safety procedures in their hospital. This finding suggests that the SSC is perceived, overall, as a complementary tool in safety procedures rather than a duplicate.

A majority of respondents expressed the opinion that the SSC was a tool which could improve communication within the teams. According to the literature, OR teams think the SSC improves the communication within the team [20], [25], [47], [50] or between interprofessional teams [30] though some teams disagree [23], and a recent systematic review of surgical team member' perceptions concluded that the SSC was positively perceived for communication [51]. However, the real impact of the introduction of the SSC on safety communication between OR teams' member remains much less evident [52].

We expected to see differences in the perceptions of the SSC between respondents working in hospital using the SSC versus those not. Some differences were found: among respondent working without SSC, the SSC was more often perceived as a waste of time, bringing no extra value to existing safety procedures and not having demonstrated its efficacy. Differences among most other items showed the same pattern of results, but were not significant, most likely because of a lack of power to detect differences due to small sample sizes (with SSC = 97, without SSC = 48). Of notable exception, respondents working with and without SSC were both not very convinced that the SSC facilitates teamwork, and eliminates the hierarchy between healthcare providers.

### Strengths and limitations

The current study did not assess barriers to the successful implementation of the SSC, a topic that has already been investigated [53]. The major limitation of our study is the low participation rate. It raises concern with the implementation and the compliance rates and with the assessment of perception agreements, which could be biased. Other Swiss studies examining the SSC [19], [31] are impeded by the same problem. We hypothesize that respondents were more interested in the SSC and our compliance rates may be overestimated. Lower than desired participation rates in surveys are common in health services research [54] and time trends suggest a decline in response rates over time [55], [56]. Nevertheless recent evidence from methodological studies indicates that non-response bias is not always systematic [57].

The topic of the present study could be potentially sensitive for some respondents and may have altered their response, causing information bias (or social desirability bias). We think that the design used for collecting data (anonymous questionnaire distributed during a congress) may have limited this effect, but it is not sure that confidentiality may affect information bias [58].

Finally, we should keep in mind that compliance with the SSC was self-reported and, thus, may have been overestimated in comparison with its actual use [59].

## Conclusion

In conclusion, the implementation of the SSC in Switzerland has been largely adopted in the majority of hospitals and clinics. The perceived compliance with the SSC and the perception of the SSC are rated positively overall. As determination of prevalence could be impeded by selection bias due to low participation rate, further research should be conducted to confirm these results.

## Supporting Information

Table S1 Attitudes towards the SSC among surgeons and anesthetists.
(DOCX)
