# Supplementary material for: Implementation of the Surgical Safety Checklist in Switzerland and Perceptions of Its Benefits: Cross-Sectional Survey
Source: PLoS One. 2014 Jul 18;9(7):e101915. doi: 10.1371/journal.pone.0101915 (PMC4103799; doi:10.1371/journal.pone.0101915)
Supplement: Table S1 — Attitudes towards the SSC among surgeons and anesthetists. (DOCX) [file pone.0101915.s001.docx]

**Table S1**  Attitudes towards the SSC among surgeons and anesthetists.

| *Proportion of “Fully agree or agree” with the following items:* | Surgeons (N=79) | Anesthetists (N=45) | p-value* |
| --- | --- | --- | --- |
| *The checklist…* | N(%) | N(%) |  |
| improves the safety of procedures | 71(89.9) | 40(88.9) | .863 |
| is a waste of time | 9(12.0) | 9(20.5) | .289 |
| improves team communication | 50(64.1) | 31(70.5) | .476 |
| brings no extra value to *existing* safety procedures already in place in my hospital/clinic | 15(20.5) | 7(16.3) | .632 |
| helps to develop a safety culture in surgical teams | 57(73.1) | 33(76.7) | .828 |
| has not demonstrated its efficacy in the scientific literature | 9(18.0) | 7(21.9) | .777 |
| facilitates teamwork | 36(47.4) | 15(35.7) | .221 |
| eliminates hierarchy between healthcare providers | 11(16.2) | 16(39.0) | .007 |

* Chi-square test or Fisher exact test if at least one cell had a frequency of 10 or less.
